# A “Nonsolvent Quenching” Strategy for 3D Printing of Polysaccharide Scaffolds with Immunoregulatory Accuracy

**DOI:** 10.1002/advs.202203236

**Published:** 2022-09-25

**Authors:** Zhencheng Liao, Yiming Niu, Zhenzhen Wang, Jiaxi Chen, Xiaoyan Sun, Lei Dong, Chunming Wang

**Affiliations:** ^1^ State Key Laboratory of Quality Research in Chinese Medicine Institute of Chinese Medicine & Department of Pharmaceutical Sciences Faculty of Health Science University of Macau Taipa Macau SAR 999078 China; ^2^ State Key Laboratory of Pharmaceutical Biotechnology School of Life Sciences Nanjing University Nanjing Jiangsu 210093 China; ^3^ Zhuhai UM Science & Technology Research Institute (ZUMRI) Hengqin Guangdong 519031 China; ^4^ Research Center for Tissue Repair and Regeneration affiliated to the Medical Innovation Research Department and 4th Medical Center PLA General Hospital and PLA Medical College 28 Fu Xing Road Beijing 100853 P. R. China

**Keywords:** 3D printing, host responses, nonsolvent reagents, polysaccharides, quenching, tissue scaffolds

## Abstract

3D printing enables the customized design of implant structures for accurately regulating host responses. However, polysaccharides, as a major biomaterial category with versatile immune activities, are typically “non‐printable” due to the collapse of their filaments extruded during printing. This challenge renders their potential as immunomodulatory scaffolds underexploited. Here, inspired by the quench hardening in metal processing, a nonsolvent quenching (NSQ) strategy is innovatively designed for the 3D printing of polysaccharides. Through rapid solvent exchanging, NSQ instantly induces surface hardening to strengthen the polysaccharide filaments upon extrusion, requiring neither chemical modification nor physical blending that alters the material properties. Tested with five polysaccharides with varying physicochemical properties, NSQ prints predesigned structures at organ‐relevant scales and a long shelf‐life over 3 months. Glucomannan scaffolds, fabricated via NSQ with different grid spacings (1.5 and 2.5 cm), induce distinct host responses upon murine subcutaneous implantation—from specific carbohydrate receptor activation to differential immunocytes accumulation and tissue matrix remodeling—as mechanistically validated in wild‐type and *Tlr2* ^−/−^ knockout mice. Overall, NSQ as a facile and generic strategy is demonstrated to fabricate polysaccharide scaffolds with improved shape fidelity, thereby potentially unmasking their accurate immunomodulatory activities for future biomaterials design.

## Graphical abstract

Inspired by “quench hardening” in metal processing, the authors develop a non‐solvent quenching (NSQ) strategy, utilizing non‐solvent to induce the surface hardening of the polysaccharide filaments upon extrusion. NSQ reinforces the filament shape and enables 3D printing of polysaccharides without the need for chemical modifications. NSQ enables customized design of polysaccharide scaffolds that can exert more accurate immunomodulatory activities.

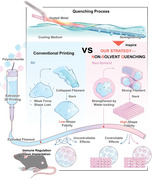

## 1 Introduction

Implantation failures due to poor tissue–biomaterials interaction, triggered by undesirable foreign body reactions (FBR), cost over USD 10 billion per year.^[^ ^1^ ^]^ Designing biomaterials to instruct cell behavior and thereby modulate FBR is of high clinical significance.^[^ ^2^, ^3^ ^]^ 3D printing, which enables the production of scaffolds with customized geometries and structures, provides a powerful tool to meet this demand.^[^ ^4^, ^5^ ^]^ However, only a limited range of biomaterials fulfills the physicochemical criteria as suitable “inks” for printing. Many other materials with diverse bioactivities are usually considered “non‐printable” under the mainstream extrusion‐based printing technology.^[^ ^6^ ^]^ For instance, polysaccharides are a major biomaterial category with inherent immunomodulatory activities; but without modification, they cannot form stable 3D structures upon extrusion, which renders their immune‐regulatory potential underexploited for biomaterial design.^[^ ^7^ ^]^

A fundamental reason behind the nonprintability of polysaccharides is their inability to maintain the shape of filaments. In a typical extrusion‐based printing procedure, a biomaterial ink is prepared in solution and extruded through a nozzle into a filament. The filament deposits onto the collector plate, stacking to a pre‐designed 3D shape while undergoing solidification (e.g., sol‐gel transition^[^ ^8^, ^9^ ^]^). At this stage, the ink must have the adequate physical strength to maintain the shape of the filaments. Nevertheless, most polysaccharides lack a sufficient inter‐molecular binding to provide this strength, and their deposited filaments collapse on the collector due to gravity before completing solidification, leading to poor shape fidelity.^[^ ^6^, ^7^ ^]^ Many strategies to overcome this challenge focus on physically blending the polysaccharide with another material^[^ ^10^, ^11^, ^12^ ^]^ or chemically modifying its sugar chains,^[^ ^13^, ^14^ ^]^ in order to reinforce the structure or accelerate solidification. Although inspiring and valuable, these approaches have introduced another material into the system or altered the chemistry of the polysaccharide, bringing in extra variables that affect host responses; they also need to balance many factors (e.g., ink strength, sol‐gel transition time, solidifying conditions) among different materials, increasing technical difficulty and narrowing the printing window.^[^ ^15^, ^16^, ^17^ ^]^ A generic method to print polysaccharides without chemical modification remains highly demanded.

**Figure 1:**
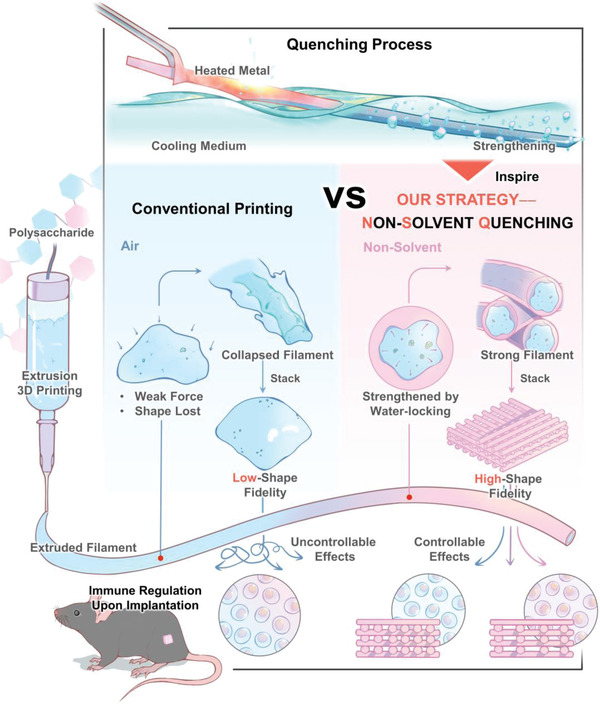
Schematic illustration of the nonsolvent quenching (NSQ) strategy for high‐fidelity 3D printing of polysaccharides without chemical modification. In conventional printing (CP) methods, when polysaccharides are extruded from the nozzle, their filaments lack adequate strength to maintain their shape and thus collapse on the collector, rendering this broad category of biomaterials “non‐printable.” Our strategy, inspired by “quench hardening” in metal processing, utilizes an appropriate non‐solvent reagent that rapidly induces the surface hardening of the polysaccharide filament upon extrusion. In this way, NSQ reinforces the filament shape and enables the formation of the pre‐designed 3D structure, without the need for chemical modifications or physical blending of other materials. By solving this common challenge, NSQ enables the customized design of polysaccharide scaffolds that can exert more accurate immunomodulatory activities upon in vivo implantation.

The key to solving this problem is reinforcing the polysaccharide filaments upon extrusion. In metallurgy, quenching is a typical and efficient process of strengthening steel or ion alloys, by submerging the hot metal in water, oil, or air for rapid cooling to achieve surface hardening.^[^ ^18^, ^19^ ^]^ Inspired by this process, we proposed a “quenching strategy” for strengthening polysaccharide filaments. Instead of heating and cooling, for polysaccharides, our strategy induces surface hardening through solvent exchanging. Since polysaccharides are prepared in aqueous solutions, we planned to screen an appropriate non‐solvent reagent^[^ ^20^ ^]^—that is miscible with water but not with the polysaccharide. As illustrated in **Figure** **1**, when the polysaccharide solution is extruded in filaments into a nonsolvent, the nonsolvent quickly replaces the water molecules at the interface. Here, the polysaccharide molecules in the outer layer of the filament immediately become supersaturated and solidify, forming a framework to lock the water inside the filament. As such, the nonsolvent “quenches” the polysaccharide filament, utilizing the force of water within the filament to maintain the structure, so that the filament can continue to stack into the pre‐designed shape without collapse. We expected this non‐solvent quenching (NSQ) strategy to serve as a generic approach to print non‐modified polysaccharides with high fidelity, thereby unmasking their subtle immune activities to modulate FBR for future implants design. To verify our hypothesis, we first screened the appropriate non‐solvent for different polysaccharides, optimized the printing conditions, and finally assessed how this method could endow polysaccharides with precise modulation of host immune responses in wild‐type and knockout mice models.

## 2 Results and Discussions

**Figure 2:**
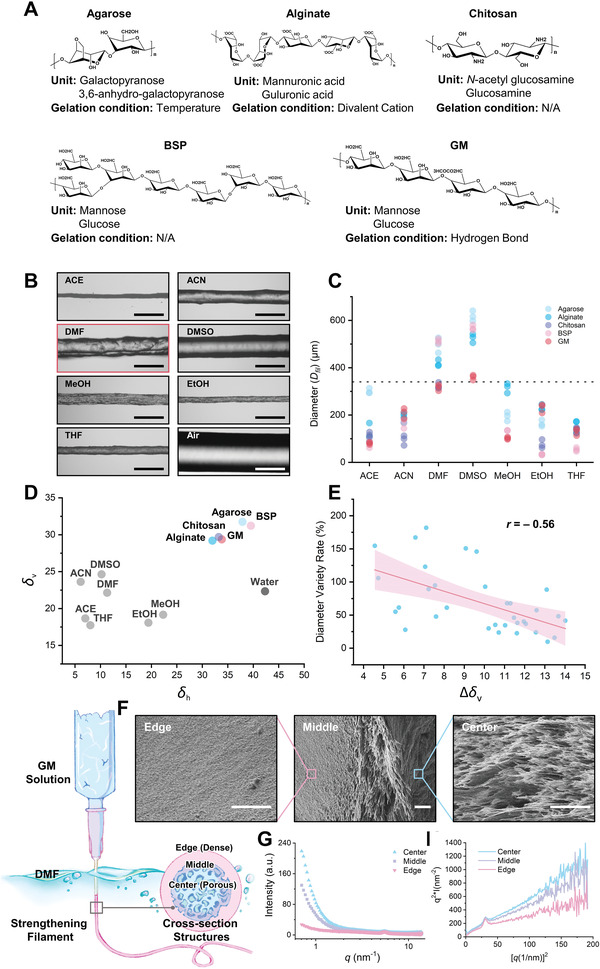
Screening and verifying non‐solvent reagents for 3D printing of polysaccharides. A) Key properties of five representative polysaccharides used for fabricating biomaterials: agarose, alginate, chitosan, *Bletilla striata* polysaccharide (BSP, a branched glucomannan), and GM (glucomannan from konjac, mostly linear). B) Images of GM filaments formed in different non‐solvent reagents. Red frame: the optimal non‐solvent for GM. C) Effect of nonsolvent reagents on the extruded filaments’ diameter. Dotted line: the y‐position of *D*_noz_. D) Solubility parameters of various polysaccharides and solvents in Bagley's 2D graph. E) The correlation between Δ*δ*_v_ and the diameter variety rate with linear regression. Pink zone: the 95% confidence intervals. F) SEM images of GM filaments’ cross‐section at different radial positions. G,H) SAXS profiles of the GM filaments at different radial positions. Scale bars: 500 µm in (B) and 5 µm in (F).

Our first step was to screen appropriate non‐solvent reagents that could maintain the filament shape of each polysaccharide through quenching. We chose five polysaccharides widely used for fabricating tissue scaffolds—i) agarose, ii) alginate, iii) chitosan, iv) *Bletilla striata* polysaccharide (BSP), and v) konjac glucomannan (GM), with representative sugar components and physical properties^[^ ^21^, ^22^, ^23^, ^24^ ^]^ (**Figure** **2**A). In a pneumatic extrusion mode of a Bioscaffolder 3.2 printer (GeSiM mbH, Germany), we extruded each polysaccharide (w/v: 2%–8%, in water) through a nozzle with a diameter (*D*_noz_) of 340 µm into each of seven organic solvents—i) acetone (ACE), ii) acetonitrile (ACN), iii) dimethylformamide (DMF), iv) dimethyl sulfoxide (DMSO), v) methanol (MeOH), vi) ethanol (EtOH), and vii) tetrahydrofuran (THF), all fulfilling the criteria as non‐reagents for polysaccharides. We photographed the filaments 30 s after extrusion and measured their diameters (*D*_fil_) (Figure 2B for GM; Figure S1, Supporting Information for other polysaccharides). The solvent, in which *D*_fil_ was the closest to *D*_noz_, was selected as the best nonsolvent for the subsequent printing. Our reason for this selection criteria was: after the filaments’ extrusion from the nozzle, a *D*_fil_ too larger than *D*_noz_ suggests the failure to lock the water inside the filament, and a *D*_fil_ far smaller than *D*_noz_ indicates distraction of the printed paths from the predesigned ones—both leading to loss of fidelity. As illustrated in Figure 2B and quantified in Figure 2C, the best nonsolvent for agarose, alginate, chitosan, BSP, and GM was ACE, MeOH, DMF, ACN, and DMF, respectively.

How could these different nonsolvent reagents perform different quenching effects to affect the filament size of each polysaccharide? We assumed that it is related to the affinity between a polysaccharide and a nonsolvent. If this affinity is too high, the polysaccharide tends to be dispersed in this nonsolvent (close to the dissolved state in the water); but, if this affinity is too low, this nonsolvent may completely repel the polysaccharide—both extremes cannot trigger the outside‐inward solidification as expected. To describe this affinity, we calculated the solubility parameter^[^ ^25^ ^]^ (SP) of each polysaccharide, each nonsolvent reagent, and water. Between a polysaccharide and a non‐reagent, a smaller difference in SP implicates a higher affinity. We plotted each polysaccharide/solvent's SP on a Bagley's 2D graph (**Table** **1** and Figure 2D), with two parameters *δ*_h_ and *δ*_v_ —where *δ*_h_ reflects hydrogen bond interactions and *δ*_v_ represents the combined contributions from polar (*δ*_p_), and dispersion (*δ*_d_).^[^ ^25^, ^26^ ^]^ Then, we measured the dot‐to‐dot distance to obtain the four Δ‐values (Δ*δ*_d_, Δ*δ*_p_, Δ*δ*_h_, and Δ*δ*_v_)—which revealed the tendency of a polysaccharide to be dissolved in one solvent against another—and fit each Δ‐value to the diameter variety rate of the polysaccharide filaments. As shown in Figure 2E, we found a negative correlation between Δ*δ*_v_ and the diameter variety rate of the polysaccharide filaments, suggesting that the dispersion and polarity of a non‐solvent could help to determine the diameter of the polysaccharide filament in this non‐solvent. Finding an appropriate level of Δ*δ*_v_ between a polysaccharide and a nonsolvent may generate a *D*_fil_ close to the *D*_noz_ of the filaments upon supersaturation, hence achieving high fidelity of printing.

**Table 1:** Solubility parameters for representative polysaccharides

Name	*δ*_d_	*δ*_p_	*δ*_h_	*δ*_v_
Agarose	21.80	23.09	37.88	31.76
Alginate	18.74	22.40	31.97	29.21
Chitosan	19.19	22.66	33.22	29.69
BSP	19.85	24.10	39.53	31.22
GM	22.26	19.17	33.81	29.38

In our NSQ strategy, the selected nonsolvent should induce inward solidification of the polysaccharide filaments from the outside. To validate this process, we employed scanning electron microscopy (SEM) and small‐angle X‐ray scattering (SAXS) to investigate the structural change of the filaments upon entering the non‐solvent. Although the nozzle size for printing is 340 µm, we purposely prepared filaments with a 1 mm diameter for better observation. We selected GM as the model material and extruded it in the form of filaments into its optimal nonsolvent reagent DMF for 30 s. As revealed by SEM (Figure 2F), the filaments had a dense shell, as a result of the polysaccharide's phase separation from water to DMF, and a porous interior (300–1000 nm). Furthermore, SAXS was performed to obtain the microstructural information of the extruded polysaccharide filaments. Since the solidified outer layer is an aggregated structure with crystal characteristics led by supersaturation, the difference in crystallinity between the interior compartments and outer ones at the initial stage could be detected. As indicated by the scattering intensity image (Figure 2G) based on SAXS data, the outer layer had a higher degree of crystallization with less porosity than did the cortex. Further analysis with a Kratky plot (Figure 2H) revealed a steeper decline in aggregation from the surface to the center of the filaments, confirming the outside‐inward solidification as designed.

**Figure 3:**
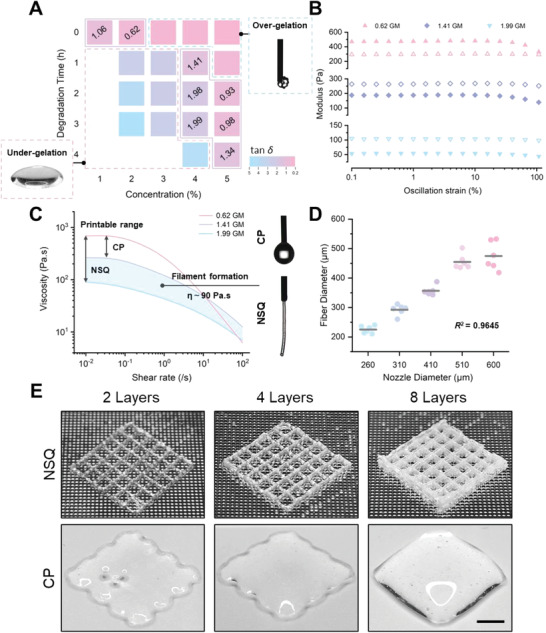
3D printing of GM via NSQ. A) Exploring the suitable printing window by generating a heat map of the tested tan *δ* values. Inset images showed the scenarios of over‐ and under‐gelation upon extrusion. B,C) Rheological characterization of printable GM, showing a broader selection range of modulus and viscosity when printed with NSQ. Filled markers: the storage moduli (*G*′); Open markers: the loss moduli (*G*″). Inset images showed the filament formation by CP and NSQ with the same viscosity (90 Pa s). D) A linear relationship between the diameter of GM filaments and that of the extrusion nozzle, when printed with NSQ (*n* = 5). E) Representative images of GM printed with NSQ or CP, in a grid structure of 2, 4, and 8 layers. Scale bar: 3 mm.

Next, we took GM as an example to demonstrate the NSQ printing. Our laboratory routinely prepared GM with different molecular weights—thus different viscosities—through enzymatic hydrolysis. Here, we used five of them to explore a suitable printing window. The preliminary tests suggested that GM inks with excessive viscosity clogged the needle while those with insufficient viscosity had low precision; the samples with a tan *δ* value (*G*″/ *G*′) between 0.62 and 1.99 were suitable for printing (**Figure** **3**A). Among them, we selected three samples with large differences in tan *δ* values and found that their modulus ranged from 53 to 469 Pa (Figure 3B) and viscosity spanned between 89 and 683 Pa s (Figure 3C). Notably, we compared the filament formation between NSQ and conventional printing (CP) to see whether it could form drops or a continuous flow upon extrusion. The result highlighted that NSQ could accommodate a wider range of materials concentrations than CP, as samples at a low viscosity maintained the filament shape when extruded into DMF. Furthermore, we found that the diameters of the fabricated filaments were close to the indicated nozzle diameter (*R* ^2^ = 0.9645, Figure 3D), providing a reference for setting printing parameters under different equipment conditions. Finally, we chose the ink with a tan *δ* value of 1.41 for the subsequent experiments. As shown in Figure 3E, GM fabricated via NSQ to 2, 4, and 8 layers in height maintained high fidelity and resolution, while the same ink printed by CP collapsed and failed to show the pre‐designed geometry. Additionally, both gross view (Figure S2A, Supporting Information) and the rheological analysis (Figure S2B, Supporting Information) illustrated that the NS treatment increased mechanical strength of GM solution, which transformed from liquid to solid with a remarkable increase in the storage modulus from 187.7 Pa to 1921.17 Pa (1023.53%). Similar results highlighting the stability and fidelity of NSQ were observed by printing a cylinder model (Movie S1, Supporting Information).

**Figure 4:**
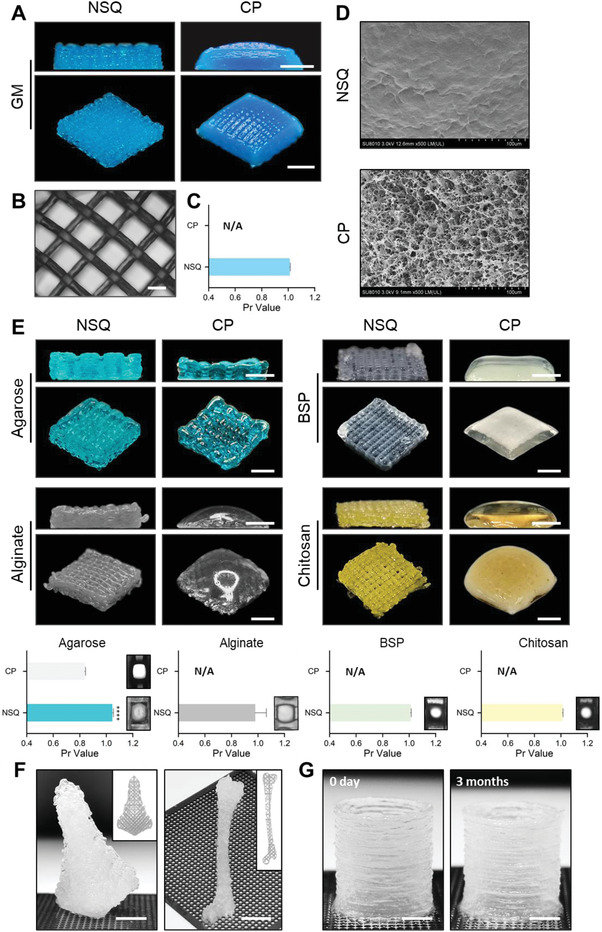
NSQ offers high fidelity and stability in printing different polysaccharides. A–D) Evaluation of the GM grid structures printed with NSQ or CP, with A) vertical and lateral view of the printed scaffolds, B) optical microscope images of grid constructs, C) calculated Pr values, and D) SEM pictures of surface structures. E) Gross view and Pr values of the grid structures of agarose, alginate, BSP, and chitosan printed by NSQ or CP. Pr values reflect the fidelity of materials. ****p < 0.0001 (*n* = 6). F) Human nose‐shaped and rat thigh bone construct fabricated via NSQ using GM. Inset images showed the printing path generated by Gesim Robotics. G) A cylinder model made from GM printed through NSQ maintained its structure after 3 months. Scale bars: 3 mm in (A, E,G), 500 µm in (B), and 5 mm in (F). Results are shown as mean ± SD. The differences between NSQ and CP groups were analyzed using an unpaired two‐tailed Student's *t‐*test.

Then, we evaluated the fidelity of polysaccharide scaffolds fabricated by NSQ. First, we calculated the printability (Pr) based on the square shape of the grids in the printed GM scaffold after alkali processing. Pr is a standard parameter to evaluate the fidelity of materials, with its value close to 1.0 reflecting the highest fidelity. Pr > 1.0 suggests the material is overconcentrated, leading to the irregular shape of the filaments after the extrusion process; Pr < 1.0 suggests a collapse of the filaments after extrusion and closure of the square on the collector. Notably, the GM scaffold fabricated through NSQ had a 1.01 ± 0.01, indicating a desirable shape fidelity using the non‐solvent strategy (**Figure** **4**A–C). Second, we compared the surface morphology of GM scaffolds fabricated through NSQ or CP, finding that the NSQ method produced a dense shell of the filaments, while the CP method made a porous one, validating our assumption of “water‐locking” reinforcement and illustrating that the non‐solvent did not affect the scaffold structure in the process of elution (Figure 4D).

To assess whether NSQ could serve as a generic approach for polysaccharide printing, we applied it to the other four polysaccharides tested (each extruded into its nonsolvent match). As expected, NSQ ensured a desirable printing quality for all of them, producing a much higher fidelity than using CP as reflected by the Pr values (Figure 4E): agarose, alginate, BSP, and chitosan, despite their different sugar components and inherent cross‐linking mechanisms (or non‐crosslinked, for the last two). The data highlight that NSQ can be applied to multiple polysaccharide types.

To test whether NSQ has the potential to fabricate complex constructs in large tissue scales, we modeled a rat thigh bone (6 × 35 × 4 mm) and a human nose (15 × 25 × 10 mm) by printing GM. The printed structures were highly stable even after stacking of 50 layers in the *z*‐direction (Figure 4F). All the individual layers and deposited filaments remained visible and faithfully followed the path created by the software. Throughout the entire NSQ procedure, we did not blend any reinforcing materials that were commonly used in conventional methods or perform any tedious cross‐linking treatments. Notably, the cylinder model (1‐cm height) printed through NSQ maintained its structure after 3 months, without any deformation (Figure 4G). This encouraging finding highlights the advantages of NSQ to print scaffolds with long shelf‐life and high stability, which are practical advantages of biomaterials toward clinical applications.

Having established NSQ as a reliable method for 3D printing of polysaccharides, we tested whether it could maximize the biological functions of the fabricated scaffolds. Taking GM as an example, it has inherently versatile activities in immunomodulation, thanks to its repeating mannose and glucose units that are naturally recognized by multiple innate immune receptors; and their activities also depend on size, assembly, and side chains, among other physicochemical factors.^[^ ^27^, ^28^ ^]^ Due to the previous hurdles in printing GM, it had been unknown whether a fine control of the 3D structure of GM scaffolds could generate specific immune responses from the host. We set out to test whether NSQ could endow GM with specific modulation of host responses in vivo.

Before in vivo studies, elemental analysis (EA, Figure S3A, Supporting Information) and gas chromatography linked to a flame‐ionization detector (GC‐FID, Figure S3B, Supporting Information) were separately employed to detect any organic solvent residue in the NSQ‐printed GM scaffolds. Both experiments showed that rinsing could effectively remove the nonsolvent agent DMF from the scaffolds, with no nitrogen and DMF detected by EA and GC‐FID, respectively. Notably, GC‐FID has a high sensitivity for detecting DMF, with a limit of detection at 0.007%, which is far below the residual solvent limit for medical devices (0.088%) stated by the International Council for Harmonisation of Technical Requirements for Pharmaceuticals for Human Use (ICH).^[^ ^29^ ^]^ Besides, in vitro studies showed that GM fabricated with NSQ had no cytotoxicity (Figure S3C,D, Supporting Information) and supported macrophage cell adhesion (Figure S3E, Supporting Information). Therefore, we considered the scaffolds free of organic solvent and safe for in vivo implantation.

**Figure 5:**
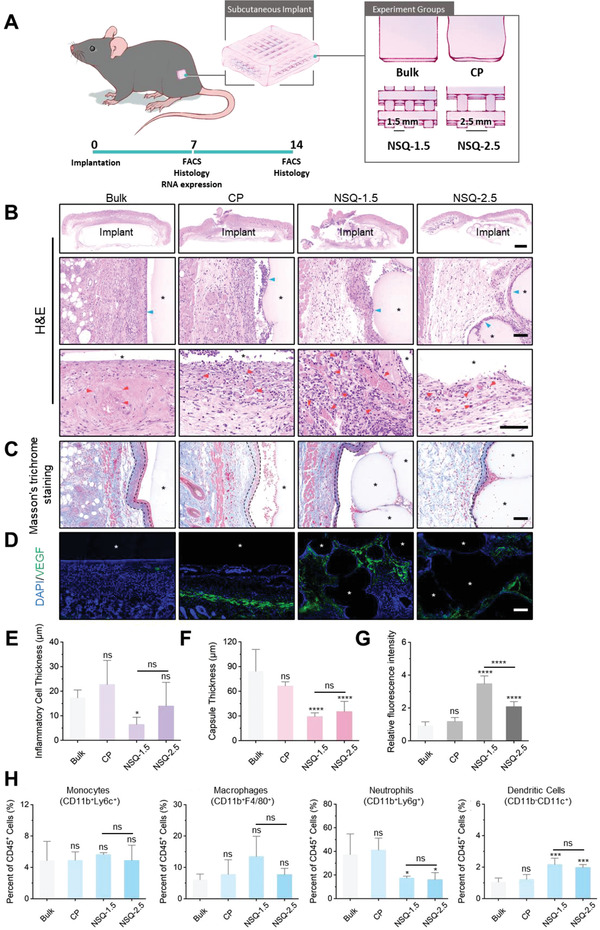
Histological and cellular analysis of FBR surrounding various GM implants. A)  Schematic illustration of the in vivo experiment: subcutaneous implantation of four GM scaffolds, fabricated via normal gelation (Bulk), CP, and in two NSQ printing parameters, followed by histological and biological analyses at days 7 and 14 postimplantation. The data from day 7 and day 14 are shown in this figure and Figure S5 (Supporting Information), respectively. B) H&E staining. Blue arrowed: inflammatory cells; red arrowed: blood vessels. C) Masson's trichrome staining. Dotted lines: thickness and location of the fibrous capsule. D) Immunofluorescence staining for VEGF (green) and nuclei (4,6‐diamidino‐2‐phenylindole [DAPI], blue). E) Quantification of inflammatory cell infiltrating thickness. *p<0.05; ns, not significant versus the bulk group (*n* = 5). F) Quantification of capsule thickness. ****p < 0.0001; ns, not significant versus the bulk group (*n* = 5). G) Quantification of relative fluorescence intensity. ****p < 0.0001; ns: not significant versus the Bulk group (*n* = 3). H) Flow cytometric analysis of myeloid cell population. *p < 0.05; ***p <0.001; ns: not significant versus the bulk group (*n* = 3). Scale bars: 1 mm in (B) first row and 100 µm (B–D). Asterisk: implants. Results are shown as mean ± SD, with one‐way ANOVA statistical analysis.

We implanted four groups of GM scaffolds subcutaneously in wild‐type C57BL/6J mice: i) bulk: nonprinted GM; ii) CP: GM scaffolds printed via the conventional method; iii) NSQ‐1.5 and iv) NSQ‐2.5: GM scaffolds printed via NSQ (with a tan *δ* at 1.41) with two line gaps at 1.5 mm and 2.5 mm, respectively (**Figure** **5**A). Especially, the in vivo implanted GM scaffold maintained the structure, as proved by their swelling ratio and Pr value (Figure S4, Supporting Information). We collected the implants with surrounding tissue on days 7 and 14 for histological, cellular, and genetic evaluation.

First, histological examinations revealed markedly different host responses at the surface of GM polysaccharide scaffolds printed in different means. H&E staining indicated varying cell infiltration and different tissue morphology around the scaffolds (Figure 5B, quantified in Figure 5E). On day 7, more inflammatory cells accumulated around the bulk and CP samples (thickness > 15 µm) than in other groups; on day 14, the inflammatory reaction intensified in these two groups (thickness >100 µm). In contrast, both NSQ‐1.5 and NSQ‐2.5 were surrounded by tissue with a thin layer of inflammatory cells at around 20 µm until day 14 (Figure S5C, Supporting Information quantified in Figure S5F, Supporting Information). Consistently, Masson's trichrome staining showed thinner fibrous capsules around NSQ‐1.5 and NSQ‐2.5 than those on bulk or CP scaffolds (Figure 5C and Figure S5D, Supporting Information; quantified in Figure 5F and Figure S5G, Supporting Information). Particularly, both staining showed that the NSQ‐1.5 scaffolds induced the highest number of neo‐formed blood vessels around the interface. Immunofluorescence (IF) staining provided further evidence, revealing the highest expression of vascular endothelial growth factor (VEGF) around NSQ‐1.5 among all groups at day 7 (Figure 5D, quantified in Figure 5G).

Next, we used flow cytometry to analyze the profiles of innate immunocytes, which play key roles in mediating host responses.^[^ ^30^, ^31^ ^]^ In the samples collected on day 7 postimplantation (Figure 5H), much more neutrophils remained around the bulk and CP scaffolds than around the two NSQ samples. As the earliest responders with a short life cycle, neutrophils persisting at day 7 might be associated with a prolonged inflammatory response.^[^ ^32^ ^]^ In contrast, more dendritic cells (DC), which are professional antigen‐presenting cells,^[^ ^33^ ^]^ were found in two NSQ groups, indicating a more actively regulated host response. In addition, the number of monocytes showed no significant differences across the groups and that of macrophages was moderately higher in the NSQ‐1.5 group than in others. The outcomes of the IF staining for the infiltrating cells surrounding the implants, including monocytes (Ly6c), macrophages (F4/80), neutrophils (Ly6g), and dendritic cells (CD11c), were consistent with the flow cytometry results (Figure S5, Supporting Information quantified in Figure S5B, Supporting Information). On day 14 (Figure S5I, Supporting Information), the levels of these myeloid cells in all groups showed no statistical difference. Compared with day 7, a higher proportion of macrophages was observed, while the proportion of the other three cells was lower.

**Figure 6:**
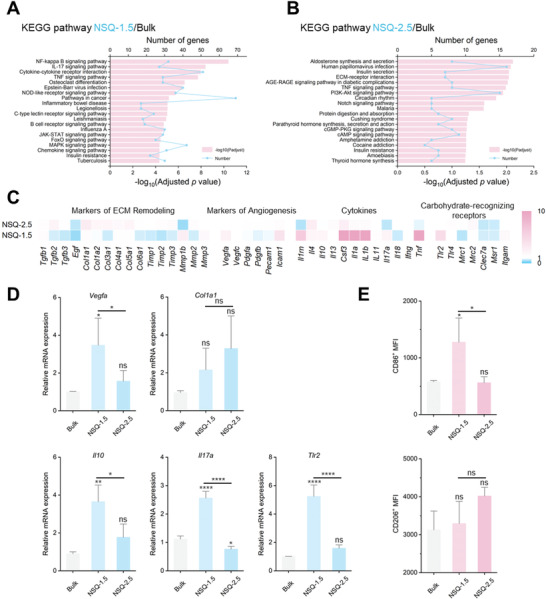
Gene analysis of tissue surrounding GM implants. Following RNA sequencing analysis of the tissue samples from four scaffolds 7 d postimplantation, KEGG pathway enrichment revealed the difference between A) NSQ‐1.5 and Bulk group and B) NSQ‐2.5 and Bulk group, and C) comparison of the expression of 41 genes in four categories, all normalized to the Bulk group. D) RT‐qPCR analysis of the levels of representative genes in agreement with the RNA‐seq data. *p < 0.05; **p < 0.01; ****p < 0.0001; ns, not significant versus the bulk group (*n* = 3). E) Expression of CD86 and CD206 surface markers on macrophages. MFI: mean fluorescence intensity. *p < 0.05; ns, not significant versus the bulk group (*n* = 3). Results are shown as mean ± SD, with one‐way ANOVA statistical analysis.

Further, to explore how such subtle geometric changes led to varying biological effects, we continued to analyze gene expression by RNA sequencing (RNA‐seq) in tissues surrounding the two NSQ scaffolds 7 d postimplantation, with the bulk group as the control. Under the condition of |log_2_ fold changes| >1 and *Q* value <0.001, the differential gene number was 3472 between NSQ‐1.5 and Bulk and 633 between NSQ‐2.5 and Bulk. The relation of these differentially expressed genes (DEGs) to key biological processes and pathways was revealed by the Kyoto Encyclopedia of Genes and Genomes (KEGG) pathway enrichment (**Figure** **6**A,B). More DEGs of NSQ‐1.5 were enriched in immune response‐associated pathways, such as NF‐kappa B signaling pathway, cytokine‐cytokine receptor interaction, and TNF signaling pathway.

We further analyzed the levels of 41 genes under four categories—i) tissue remodeling, ii) angiogenesis, iii) cytokines, and iv) carbohydrate‐recognition receptors (Figure 6C)—in reverse order to the dynamic process of host response,^[^ ^34^, ^35^ ^]^ initiating from ligand recognition, triggering inflammation (cytokine release) and angiogenesis, and leading to tissue remodeling. First, genes associated with ECM remodeling, including collagens, tissue inhibitors of metalloproteinases, and matrix metalloproteinases, were in elevated levels in NSQ‐2.5 and decreased levels in NSQ‐1.5, in agreement with the KEGG data. Second, *Vegfa* and *Icam1*, two important genes for angiogenesis, were significantly up‐regulated in NSQ‐1.5 (2.43 and 3.81 folds, respectively, versus those in Bulk). This finding was consistent with the observation in histological and IF staining and highlighted NSQ‐1.5's effect on promoting angiogenesis. Third, both anti‐inflammatory (*Il1rn* and *Csf3*) and proinflammatory (*Il1a, Il1b*, and *Tnf*) were considerably increased (6.31, 34.89, 65.74, 18.91, and 15.98 folds, respectively, versus bulk group), indicating a stronger effect of host responses by NSQ‐1.5 through a more sophisticated immune modulation. Finally, we looked at the changes in carbohydrate‐recognizing receptors, which are directly correlated with the immunoregulatory effects of polysaccharides.^[^ ^27^, ^36^, ^37^ ^]^ Intriguingly, the level of *Tlr2*, a key pattern recognition receptor that plays a pivotal role in triggering an innate immune response to mannan‐/glucan‐containing signals, increased in NSQ‐1.5 by 4.39 folds.

Additional quantitative PCR analysis (Figure 6D) verified the above RNA‐seq data, highlighting up‐regulated expressions of *Vegfa* (angiogenic), *Il10* (anti‐inflammatory), *Il17a* (proinflammatory), and, intriguingly, *Tlr2* (carbohydrate‐recognizing receptor marker) in NSQ‐1.5. Also, flow cytometry analysis (Figure 6E) showed more CD86^+^ macrophages and fewer CD206^+^ macrophages in NSQ‐1.5 than in NSQ‐2.5, in agreement with the RNA‐seq data—higher *Tlr2* and *Mrc* (both 1 and 2) levels in NSQ‐1.5 and NSQ‐2.5, respectively.

Since the mechanical change of biomaterials may also affect immune responses, we tested the local mechanical properties of NSQ‐1.5 and NSQ‐2.5 by calculating their normalized storage modulus, which showed no significant difference (Figure S6A, Supporting Information). Further, we found similar mechanical signals resulting from NSQ‐1.5 and NSQ‐2.5 by analyzing the transcriptional expression of three typical makers of biomechanical pathways,^[^ ^38^, ^39^ ^]^ YAP (Yes‐associated protein), TAZ (transcriptional coactivator with PDZ‐binding motif) and YAP/TAZ target genes (Figure S6B, Supporting Information), which had no significant differences between the NSQ samples. Such evidence excluded mechanical properties as a major reason affecting the observed host responses.

**Figure 7:**
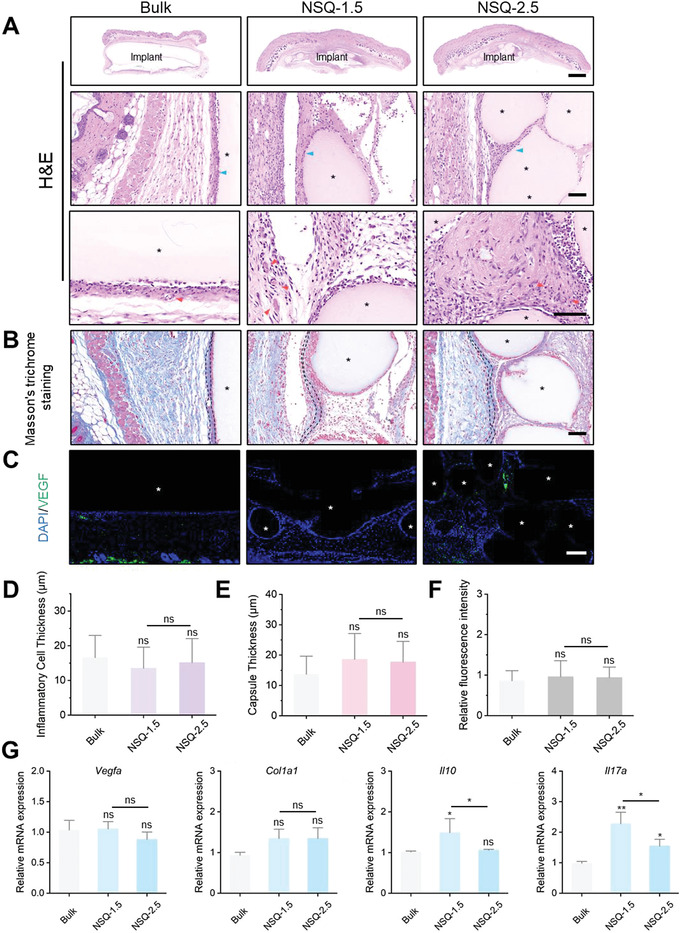
Analysis of FBR surrounding GM implants in *Tlr2* ^−/−^ KO mice. A) H&E staining. Blue arrow: inflammatory cells; red arrow: blood vessels. B) Masson's trichrome staining. Dotted lines: thickness and location of the fibrous capsule. C) IF staining for VEGF (green) and nuclei (4,6‐diamidino‐2‐phenylindole [DAPI], blue). D) Quantification of the inflammatory cell infiltration thickness; ns: not significant versus the bulk group (*n* = 5). E) Quantification of capsule thickness; ns, not significant versus the bulk group (*n* = 5). F) Quantification of relative fluorescence intensity; ns: not significant versus the bulk group (*n* = 3). G) RT‐qPCR analysis of the levels of representative genes. *p < 0.05; **p < 0.01; ns: not significant versus the Bulk group (*n* = 3). Scale bars: 1 mm in (A) first row and 100 µm (A–C). Asterisk: implants. Results are shown as mean ± SD, with one‐way ANOVA statistical analysis.

The intriguing finding of TLR2 involvement suggested that this receptor might play a significant impact in differentiating the host responses to the two types of 3D printed GM scaffolds. Then, we employed *Tlr2* ^−/−^ knockout (KO) mice model and repeated the implantation procedure with the two NSQ and bulk scaffolds, with a focus on host responses 7 d postimplantation. Interestingly, the previously observed difference in histology across different groups was eliminated, as both H&E (**Figure** **7**A) and Masson's staining (Figure 7B) showed similar profiles of immunocytes filtration (average inflammatory cell thickness: 13–17 µm) (Figure 7D) and collagen deposition (average capsule thickness: 13–19 µm) (Figure 7E) in all three groups. Notably, neo‐vessels around NSQ‐1.5 in the KO mice were far fewer than in the wild‐type mice and became comparable with other groups, accompanied by the expression of *Vegfa* at a similar level among all groups (Figure 7C, quantified in Figure 7F). Likewise, the differences in the expression of four key genes were narrower, with two (*Vegfa* and *Col1a1*) with no significant differences among the groups (Figure 7G). These findings suggested that, in the absence of TLR2, the different host responses to different GM scaffolds did not exist, confirming the importance of this carbohydrate‐binding receptor in mediating this process.

The above analyses at histological, cellular, and genetic levels suggested that the same GM polysaccharide printed in different settings generated distinct FBR in the host tissue. These findings verified our hypothesis that enabling high‐fidelity 3D printing of polysaccharides could unmask subtle and more accurate immunomodulatory activities from these molecules. Among various natural and synthetic materials types, polysaccharides are a special source for their inherent and rich immune activities, because innate immune cells express a plethora of carbohydrate receptors to recognize various glycan‐containing structures, consequently triggering diverse pro‐/anti‐inflammatory responses.^[^ ^40^, ^41^ ^]^ Even if with the same backbone, as both ours^[^ ^27^, ^42^ ^]^ and other studies^[^ ^43^, ^44^ ^]^ have discovered, polysaccharides in different sizes, scales, or assemblies could exhibit varying immune activities. Here, the NSQ strategy bridges a long‐existing gap to make polysaccharides—traditionally considered “non‐printable”—become printable, hence offering vast possibilities to unveil their full immune activities that have not been exploited.

In addition, the different FBR to the scaffolds were not superficial phenomena but involved different types and extents of receptor activation, inflammation, as well as tissue matrix reorganization. The two geometries well‐defined through NSQ triggered differential host responses that were not observed in Bulk or CP‐prepared GM scaffold. Between them, NSQ‐1.5 had a higher impact on promoting angiogenesis while NSQ‐2.5 more directly affected ECM remodeling, with a comprehensive set of differences in cell receptor expression, cytokine release, and immune cell phenotypes around. Our findings add weight to the recent literature on the importance of defining physical cues of biomaterials (e.g., mechanical properties,^[^ ^45^, ^46^ ^]^ geometries,^[^ ^47^ ^]^ structure,^[^ ^48^ ^]^ etc.) for modulating immune responses. Although this study focuses on this new methodology and does not target a specific type of tissue repair or a particular disease, it demonstrates the potential of NSQ for achieving more controllable FBR modulation in varying directions in customized implant design.

Finally, to examine whether NSQ could provide alternative advantages in unmasking the inherent immunomodulatory activities from unmodified polysaccharides, we performed a chemical modification of GM and printed the scaffolds via CP for in vivo implantation. We generated acetyl esters of GM into GMAC, which represents common modifying methods to make polysaccharides “printable”—by increasing hydrophobic moieties^[^ ^49^, ^50^ ^]^—and is also convenient and relatively consistent. Indeed, esterification provided GMAC with excellent physical properties to be directly printed by CP, followed by the fabrication of grid structures with two spacing (GMAC‐1.5 and GMAC‐2.5) and subcutaneous implantation in mice—all conditions likewise in the GM samples processed by NSQ.

Nevertheless, after 7 d postimplantation, the chemically modified samples showed excessive inflammation (Figure S7A, Supporting Information quantified in Figure S7D, Supporting Information) and thick fibrous encapsulation (Figure S7B, Supporting Information quantified in Figure S7E, Supporting Information). Between the GMAC‐1.5 and GMAC‐2.5 scaffolds, the fluorescence intensity of *Vegfa* (Figure S7C, Supporting Information quantified in Figure S7F, Supporting Information) and the expression levels of *Col1a1*, *Il10*, and *Il17a* had no significant differences (Figure S7G, Supporting Information). Further, we compared the results between GMAC and GM groups. First, the magnified inset images in Figure S7A (Supporting Information) highlighted persisted inflammation and impaired wound healing in both GMAC‐1.5 and GMAC‐2.5 groups; while in the NSQ‐printed GM groups, the wounds of all samples had entered the remodeling phase. Second, GMAC scaffolds triggered obviously stronger inflammatory reactions in the surrounding tissue than did GM samples—evidenced by the denser layer of inflammatory cells (≈23 µm for GMAC vs ≈15 µm for GM). Third, for GM samples of NSQ‐1.5 and NSQ‐2.5, the different grid spacing led to different profiles in the thickness of cell infiltration and fibrous encapsulation, as well as relative fluorescence intensity of *Vegfa*; but, these differences disappeared between the GMAC samples. Further analysis revealed more peri‐implant CD86^+^ macrophages (Figure S7H, Supporting Information) and markedly higher expression of pro‐inflammatory genes (*Tnf* and *Nos2*, Figure S7I, Supporting Information) in all chemically modified GMAC samples than in non‐modified GM samples. These data suggested that the chemically modified samples with spacing 1.5 and 2.5 failed to exhibit the subtle immunomodulatory accuracy of GM that could be exhibited in NSQ‐printed samples. Instead, both GMAC samples exerted excessively strong stimulations with no differences.

The above findings also implicated that a chemical modifying method, despite being able to convert polysaccharides into a printable derivative, might easily change or mask the inherent properties of the polysaccharide. This was one of the main motivations we developed methods avoiding chemical modifications to print polysaccharides at the very beginning. In comparison, NSQ could help to retain polysaccharides’ inherent properties, without introducing other (perhaps more dominant) factors brought by modification, and may contribute a new alternative for printing carbohydrate materials with more accurate immunomodulatory activities.

A fundamental innovation in developing NSQ is the introduction of “quenching” from metal processing into polysaccharide printing. This inspiration has brought in two major advantages to NSQ. First, because this quenching relies on solvent exchanging, NSQ does not require chemical modifications that change molecular structures or harsh conditions (e.g., extreme temperatures or pHs) that may break the sugar chain. Thus, it keeps polysaccharides’ original features to the maximum during fabrication. Second, because this quenching utilizes the “locked” water molecules to strengthen the polysaccharide filaments, several other factors (e.g., mechanical properties of the polysaccharide ^[^ ^6^, ^51^ ^]^ or cross‐linking kinetics^[^ ^51^ ^]^) that used to be crucial for real‐time providing mechanical support in conventional printing are no longer critical. Researchers can skip the process of multiparameter coordination while only needing to focus on basic printing settings.

Our data highlighted NSQ as a generalizable strategy for polysaccharide printing with high application potential. As tested, NSQ efficiently printed five polysaccharides—across different components, charges, and gelling properties—into stable scaffolds with high fidelity. In addition, the nonsolvent agents used are easy to remove (as demonstrated by cytotoxicity studies), and the formed materials could be stored in the nonsolvent for several months until collection, which is both practical advantages for further application of this technology. Our strategy also echoes with other recent innovations. For instance, Feinberg et al. invented an excellent method (“FRESH”: freeform reversible embedding of suspended hydrogels) to improve the printing fidelity of collagen and other materials, by setting up a thermo‐reversible support bath made of gelatin.^[^ ^52^, ^53^ ^]^ Despite our different approaches and target materials, both strategies focus on controlling the interface between the inks and the environment, which can complement each other in certain applications. Moreover, the NSQ‐printed scaffolds represent a “pseudo‐hydrogel” state—they can provide mechanical support as hydrogels do, while being able to re‐disperse in the water (which hydrogels do not). This feature is particularly suitable for fabricating sacrificial scaffolds, which many types of approaches (either or not based on extrusion) need for providing in‐time support to other building‐block materials.^[^ ^54^ ^]^ Nevertheless, more applications of NSQ in combined use with nonextrusion methods, e.g., stereolithography (SLA) and digital light processing (DLP), for biological printing needs future exploration.

The limitations of this study, which also motivate our future investigations, are in at least two aspects. First, we calculated the solubility parameter to predict how non‐solvent reagents could control the filament sizes, and our fitting in the given examples was successful; however, for wider applications, we must consider that: i) not all materials can be easily calculated for their solubility parameters due to the structural complexity and other unclear factors of polymers; ii) even for the same polysaccharide, its linear/branching degree, molecular size, or spontaneous assembly could affect our prediction. More high‐throughput physics simulation, ideally seeking help from artificial intelligence learning, might provide a constantly evolving tool for the selection of the appropriate non‐solvent (or multi‐nonsolvent formula). Second, although we identified TLR2 as a key receptor differentially activated between two NSQ‐printed GM scaffolds, we also noticed the up/down‐regulation of other carbohydrate receptors and immunomodulatory signaling pathways. The molecular mechanisms underlying cell‐scaffold interactions, including the types of receptors involved in, their crosstalk in the cells, as well as the kinetics of glycan–receptor interactions, remain intriguing questions for future studies.

## 3 Conclusion

In summary, we have demonstrated a facile and generic strategy for high‐fidelity 3D printing of polysaccharides with varying physicochemical characteristics. Based on an innovative non‐solvent quenching (NSQ) method, this strategy requires no chemical modification or physical blending, and can efficiently fabricate polysaccharides into sophisticated structures with high shape fidelity at organ‐relevant scales and a longer shelf‐life. Furthermore, using a linear immunoactive polysaccharide (konjac glucomannan, GM) as an example, NSQ fabricated scaffolds with different grid spacing (1.5 and 2.5 cm, respectively) revealed distinct immuno‐regulatory effects, mediated by differential activations of carbohydrate receptors (notably TLR2) and inflammatory cascades in mice, which lead to different tissue matrix organization around the implants. Overall, this method enables the design of polysaccharide‐based immunomodulatory scaffolds with well‐defined structural properties through 3D printing, demonstrating how the customized design could exhibit differential (but previously underestimated) immune activities in vivo. Given the rich sources and versatile bioactivities of polysaccharides (one of the most applied biomaterials categories)—in addition to the long‐term challenge in fully fabricating them with 3D printing, our strategy may open new avenues for more accurately understanding and controlling tissue‐implants interactions in a broad range of therapeutic applications.

## 4 Experimental Section

### Chemicals and Reagents

Konjac glucomannan (GM, 102 800 mPa s) was provided by Shimizu Chemical (Japan). Other polysaccharides and chemical reagents were purchased from Aladdin (China). Fetal bovine serum and cell culture medium was obtained from Life Technologies. Calcium AM/PI kit was purchased from Shanghai Yisheng (China). GoTaq 2‐Step RT‐qPCR system was purchased from Promega. All primers used for RT‐qPCR were synthesized by Life Technologies (China), and their sequences are listed in Table S1 (Supporting Information). The antibodies used in this study are listed in Table S2 (Supporting Information).

### Nonsolvent Reagents Screening

A syringe containing the polysaccharide solution was loaded onto the Bioscaffolder 3.2 printer. For all measurements, a 23‐gauge needle was used. Each sample was extruded directly into the nonsolvent reagents under minimum pneumatic pressure. Thirty seconds after extrusion, the filament was photographed under an optical microscope and the diameter was measured by ImageJ.

### Estimation of Solubility Parameter by Hoy's System^[^ ^55^ ^]^

Hoy's method had been published to predict the solubility parameter components. Tables S2–S5 (Supporting Information) lists constants for three additive molar functions: molar attraction constant (*F*_t_), polar components (*F*_p_), and Lydersen correction (Δ*_T_* ^(P)^) for polymer. These values are to be used in auxiliary equations and the expression for the overall solubility parameter (*δ*_t_). Hoy determined nonpolar, polar, and H‐bonding parameters by semi‐empirical methods which involved:

a) Calculation of the H‐bonding parameter from
(1) $$\delta_{h}=\delta_{t}{\left[\left(\alpha^{\left(P\right)}-1\right)/\alpha^{\left(P\right)}\right]}^{1/2}$$
b) Calculation of the polar parameter from
(2) $$\delta_{p}=\delta_{t}{\left(\frac{1}{\alpha^{\left(P\right)}}\frac{F_{p}}{F_{t}+B/\bar{n}}\right)}^{1/2}$$
c) Calculation of the nonpolar parameter from
(3) $$\delta_{d}={\left(\delta_{t}^{2}-\delta_{p}^{2}-\delta_{h}^{2}\right)}^{1/2}$$
d) The final expression for *δ_t_*_(total)_
(4) $$\delta_{t}=\left(F_{i}+B/\bar{n}\right)/V$$
e) Calculation of the combined contributions from polar and dispersion from
(5) $$\delta_{v}={\left(\delta_{d}^{2}+\delta_{p}^{2}\right)}^{1/2}$$
f) A number of auxiliary equations
(6) $$\alpha \left(P\right)=777\Sigma \Delta_{T}^{\;(P)}/V$$
  and
(7) $$n=0.5/\Delta_{T}^{\;(P)}$$

where *α* is the molecular aggregation number, describing the association of the molecules, 777 is a constant, and *V* is molar volume; n is the number of repeating units per effective chain segment of the polymer.

The calculated solubility parameters for representative polysaccharides were given in Table 1.

### Scanning Electron Microscopy (SEM)

GM filaments or scaffolds were frozen in liquid nitrogen and lyophilized post‐fabrication. The freeze‐dried sample was cut using a razor blade to make the cross‐section exposed, and subsequently sputter‐coated with Pt. Samples were imaged with SEM (SU8018, Hitachi, JPA) using an accelerating voltage of 3 kV.

### Small Angle X‐Ray Scattering (SAXS)

GM solution was extruded through a 1 mm tube into DMF and left for 30 s to obtain a filament. Three positions from the edge to the center of this filament were selected for study. Experiments were conducted on Xeuss 2.0 SAXS system (Xenocs, France) with a Pilatus 300K (Dectris) detector using Cu K*α* radiation (50 kV, 60 mA) and an exposure time of 10 min. The sample‐detector distance was 294 mm, which was calibrated using silver behenate. 1D scattering profiles were reduced from the 2D data using Foxtrot.

### Rheological Characterization

Rheological testing was carried out on a TA Instruments DHR‐2 Rheometer, using a 20 mm measuring plate. Amplitude sweeps and shear rate sweeps were performed at room temperature. The amplitude sweep was carried out at 1 Hz in a strain range from 0.1% to 100%. And the shear rate sweep was performed to measure viscosity with a shear rate varied from 0.001 to 100 s^−1^.

### Semiquantification of Printability^[^ ^16^ ^]^

When the ink was in an ideal gelation condition, the extruded filament would result into regular grids and square holes in the fabricated constructs. When the ink was in an under‐gelation condition, the extruded filament would fuse with the lower layer, thus creating approximately circular holes. And printability (Pr) of ink is based on square shape using the following function:

(8) $$\mathrm{Pr}=L^{2}/16A$$

where *L* means perimeter and *A* means area. These two values were calculated by ImageJ via optical microscope images.

### 3D Printing

Polysaccharides were dissolved and loaded into a print cartridge equipped with 23‐gauge disposable needle. The cartridge was loaded to the Bioscaffolder 3.2 printer (GeSiM mbH, Germany). The detailed printing parameters for individual inks can be found in Table S4 (Supporting Information). After printing, the printed constructs were treated with their corresponding gelation conditions if they have. the cross‐linked GM constructs were then washed with PBS thrice for further use.

### Residual Nonsolvent Testing

Elemental analysis: The elemental composition of samples was analyzed with a CHN analyzer (Vario Micro Cube, Germany).

Gas chromatography (GC) analysis: Samples subjected to GC analysis was performed on an Agilent 7890A instrument (Agilent, USA) equipped with an Agilent DB‐624 column (30 m  ×  250 µm  ×  1.4 µm) and a flame‐ionization detector (FID). The operation was performed under the following conditions: N_2_: 1.0 mL min^‐1^; injection temperature: 260 °C; detector temperature: 260 °C; column temperature programmed: increased from 40 °C to 260 °C at 15 °C min^‐1^ and held for 1 min at 260 °C.

### Cytocompatibility Tests

Raw 264.7 murine macrophages and human umbilical vein endothelial cells (HUVECs) were purchased from the ATCC (American Type Culture Collection). Cells were cultured with DMEM and 10% fetal bovine serum in a 5% CO_2_ incubator at 37 °C. 1) Cell viability assay: For toxicity assay, the culture medium was replaced with the medium extracted from the scaffold after 24 h of incubation, and the untreated medium was used as a blank control. The cell viability was examined with CCK‐8 assay on days 1, 2, and 3 after seeding. At each time point, each well 10 µL working solution of WST‐8 (5 mg mL^‐1^) was added and incubated for 2 h. The optical density value was determined at 450 nm. 2) Live/dead staining: Before seeding cells, scaffolds were sterilized by UV. Raw cells were cultured on the GM scaffold at a density of 100 000 cells per cm^2^. These samples were incubated for 24 h at 37 °C, and then washed with 1 × PBS to remove detached cells. Calcium AM/PI reagent was added into plate wells and incubated for 30 min at 37 °C followed by washing thrice with 1 × PBS. The image of adhesive cells was captured by the confocal microscope (SP8, Leica, Germany).

### Swelling Ratio Tests

The swelling ratio was determined using a classical gravimetric method. GM scaffold was immersed in 1 × PBS at 37 °C until the swelling reached equilibrium. For every time interval, the swollen scaffold was weighed, and the equilibrium swelling ratio by weight was calculated based on the following equation:

(9) $$\mathrm{Swelling}\;\mathrm{ratio}=\left(W_{s}-W_{d}\right)/W_{d}\times 100\%$$

where, *W*_s_ is the weight of swollen scaffold, and *W*_d_ is the initial weight of the scaffold after rinsing.

### In Vivo Implantation

The murine dorsal subcutaneous pocket model was selected to evaluate the foreign body reactions (FBR) of GM scaffolds. The animal experiments were carried out based on protocols approved by the University of Macau Animal Ethics Committee (Approval No. UMARE‐006‐2022 and UMARE‐037‐2017) and Nanjing University (IACUC‐2205001). 6‐9 weeks old female C57BL/6J WT and *Tlr2* ^−/−^ knockout mice were anesthetized using pentobarbital (70 mg kg^‐1^) and shaved. After sterilization with 75% ethanol, two independent incisions were created subcutaneously on the back of each mouse. After implantation, the incisions were sewn up. The experimental animals were raised according to the standard protocol.

### Histological Analysis

At each time point, mice were sacrificed and the implants along with the surrounding tissue were collected and fixed in 4% formalin, followed by dehydration before embedding in paraffin. These samples were cross‐sectioned into 6 µm for histological analysis. The sections were deparaffinized and rehydrated for hematoxylin eosin, Masson's trichrome staining. For immunofluorescent staining, the deparaffinized and rehydrated sections were blocked with bovine serum albumin and stained by an anti‐VEGF, anti‐Ly6c, anti‐F4/80, anti‐Ly6g, anti‐CD11c, and anti‐TLR2 antibody followed by 4,6‐diamidino‐2‐phenylindole (DAPI) for nuclear staining. All immunofluorescent staining was captured with a confocal microscope (SP8, Leica, Germany). The others were recorded by a light microscope (BX51, Olympus). ImageJ software (http://rsb.info.nih.gov/ij/) was utilized to quantify the inflammatory cell thickness, capsule thickness, and the fluorescence intensity of VEGF.

### Flow Cytometry

After 1‐ or 2 weeks postsurgery, the implants along with the skin tissue were collected and cut up into small pieces before digestion in 0.5 mg mL^‐1^ Liberase TL (Sigma) and 0.2 mg mL^‐1^ DNase I (Roche) in RPMI media, followed by shaking under 37 °C for 45 min. Digested samples were filtered via a 70 µm cell strainer and washed thrice with 1 × PBS. Cells isolated from the fibrous capsules were first stained with a fixable viability dye (BD Horizon) for 30 min on ice, followed by a myeloid antibody panel at 4 °C for 45 min. Then, the staining cells were fixed for 20 min at 4 °C and stored in 1 × PBS until analysis on the BD LSRFortessa Flow Cytometer (BD Biosciences). Data were analyzed using FlowJo.

### RNA‐seq and Data Analysis

RNA was extracted using TRIzol Reagent (Ambion, Life Technologies) and primed with Oligo (dT)_15_ and Random Primers for cDNA synthesis using GoScript Reverse Transcription kit guided by the manufacturer's instructions. RNA‐seq library was sequenced on the Illumina Novaseq 6000. Microarray data were analyzed on the online platform, Majorbio Cloud Platform (www.majorbio.com).

### Quantitative Real‐Time PCR (qPCR) Assay

Real‐time qPCR was performed with GoTaq qPCR Master Mix reagent and Mx3005P qPCR System following the Manufacturer's guide. This assay was accomplished in triplicates.

### Statistical Analysis

Statistical differences among samples were studied through the *t*‐test for differences between two groups, or the one‐way analysis of variance (ANOVA) with Tukey's multiple comparisons test for more than two variables using SPSS v23.0 (SPSS Inc., Chicago, USA). If appropriate, analyze by repeated measures ANOVA test with post‐hoc Bonferroni correction. The data presented as the mean ± standard deviation (SD) was obtained based on at least three independent replicates. Significance was set to *p* < 0.05. (*p < 0.05, **p < 0.01, ***p < 0.001, ****p < 0.0001).

## Conflict of Interest

The authors declare no conflict of interest.

## Supporting information

Supporting Information

Supplemental Movie 1

## Data Availability

The data that support the findings of this study are available from the corresponding author upon reasonable request.
