# Supplementary material for: A “Nonsolvent Quenching” Strategy for 3D Printing of Polysaccharide Scaffolds with Immunoregulatory Accuracy
Source: Adv Sci (Weinh). 2022 Sep 25;9(34):2203236. doi: 10.1002/advs.202203236 (PMC9731704; doi:10.1002/advs.202203236)
Supplement: Supplementary file 1 — Supporting Information [file ADVS-9-2203236-s002.pdf]

ADVANCED SCIENCE
Open Access

Supporting Information

for *Adv. Sci.*, DOI 10.1002/advs.202203236

A “Nonsolvent Quenching” Strategy for 3D Printing of Polysaccharide Scaffolds with Immunoregulatory Accuracy

Zhencheng Liao, Yiming Niu, Zhenzhen Wang, Jiaxi Chen, Xiaoyan Sun, Lei Dong* and Chunming Wang**

Supporting Information

A "non-solvent quenching" strategy for 3D printing of polysaccharide scaffolds with immunoregulatory accuracy

Zhencheng Liao, Yiming Niu, Zhenzhen Wang, Jiaxi Chen, Xiaoyan Sun, Lei Dong*, Chunming Wang**

Z. Liao, Dr. Y. Niu, J. Chen, Prof. C. Wang
State Key Laboratory of Quality Research in Chinese Medicine, Institute of Chinese Medicine & Department of Pharmaceutical Sciences, Faculty of Health Science, University of Macau, Taipa, Macau SAR
E-mail: cmwang@umac.mo

Dr. Z. Wang, Prof. L. Dong
State Key Laboratory of Pharmaceutical Biotechnology, School of Life Sciences, Nanjing University, Nanjing, Jiangsu, 210093, China
E-mail: leidong@nju.edu.cn

Prof. X. Sun
Research Center for Tissue Repair and Regeneration affiliated to the Medical Innovation Research Department and 4th Medical Center, PLA General Hospital and PLA Medical College, 28 Fu Xing Road, Beijing 100853, P. R. China
E-mail: yanzisun1979@sina.com

J. Chen, Prof. C. Wang
Zhuhai UM Science & Technology Research Institute (ZUMRI), Hengqin, Guangdong, China

Table S1. Primer sequences used for RT-qPCR in this study.

Gene	Forward (5'-3')	Reverse (5'-3')
m *Actb*	GCTGGTCGTCGACAACGGCTC	CAAACATGATCTGGGTCATCTTTTC
m *Yap1*	TACTGATGCAGGTACTGCGG	TCAGGGATCTCAAAGGAGGAC
m *Tead1*	AGCCAGATACATCAAACTCAGGACG	CTTAATGGCGGCTTGAATTTCTCGAAC
m *Ankrd1*	AGACTCCTTCAGCCAACATGATG	CTCTCCATCTCTGAAATCCTCAGG
m *Vegfa*	GTTCAGAGCGGAGAAAGCAT	TCACATCTGCAAGTACGTTCG
m *Col1a1*	TGTGTTCCCTACTCAGCCGTCT	CATCGGTCATGCTCTCTCCAA
m *Il10*	GCTCTTACTGACTGGCATGAG	CGCAGCTCTAGGAGCATGTG
m *Il17a*	TCTCCACCGCAATGAAGACC	CACACCCACCAGCATCTTCT
m *Tlr2*	TTGCTCCTGCGAACTCCTAT	CAATGGGAATCCTGCTCACT
m *Tnf*	ACGGCATGGATCTCAAAGAC	AGATAGCAAATCGGCTGACG
m *Nos2*	CCAAGCCCTCACCTACTTCC	CTCTGAGGGCTGACACAAGG

Table S2. The antibodies used in this study.

Antigen	Fluorophore	Manufacturer	Dilutions
CD45	PerCP/Cy5.5	Biolegend	1:100
CD11b	FITC	Biolegend	1:100
CD11c	PE/Cy7	Biolegend	1:100
Ly6c	APC	Biolegend	1:100
Ly6g	PE	Biolegend	1:100
F4/80	APC	Biolegend	1:100
CD86	PE	Biolegend	1:100
CD206	PE/Cy7	Biolegend	1:100

Table S3. Solubility parameter component group contributions for representative polysaccharides proposed by Hoy's system.

Structural group	No. groups	$F_{t,i}$ ($J^{1/2}cm^{3/2}mol^{-1}$)	$F_{p,i}$ ($J^{1/2}cm^{3/2}mol^{-1}$)	$\Delta_{T,i}^{(P)}$	V_i (cm^3mol^{-1})
**Agarose**					
-CH2	1	269	0	0.02	16.1
-CH2 in ring	1	269	0	0.02 (×2/3)	16.1
-CH	10	176	0	0.013 (×2/3)	-1
-OH (prim.)	1	675	675	0.049	10
-OH (second.)	3	591	591	0.049	13
-O- (acetal)	2	236	102	0.018	3.8
-O- (acetal) in ring	2	236	102	0.018 (×2/3)	3.8
-O- (ether) in ring	1	235	216	0.018 (×2/3)	3.8
6-membered ring	2	-48	61	-0.0035	16
**Alginate**					
-CH	25	176	0	0.013 (×2/3)	-1
-OH (second.)	10	591	591	0.049	13
-O- (acetal)	5	236	102	0.018	3.8
-O- (acetal) in ring	5	236	102	0.018 (×2/3)	3.8
-COOH	5	565	415	0.039	28.5
6-membered ring	5	-48	61	-0.0035	16
**Chitosan**					
-CH2	2	269	0	0.02	16.1
-CH	10	176	0	0.013 (×2/3)	-1
-OH (prim.)	2	675	675	0.049	10
-OH (second.)	2	591	591	0.049	13
-O- (acetal)	2	236	102	0.018	3.8
-O- (acetal) in ring	2	236	102	0.018 (×2/3)	3.8
-NH2	2	464	464	0.035	19.2
6-membered ring	2	-48	61	-0.0035	16
**BSP**					
-CH2	7	269	0	0.02	16.1

-CH	35	176	0	0.013 (×2/3)	-1
-OH (prim.)	7	675	675	0.049	10
-OH (second.)	14	591	591	0.049	13
-O- (acetal)	7	236	102	0.018	3.8
-O- (acetal) in ring	7	236	102	0.018 (×2/3)	3.8
6-membered ring	7	-48	61	-0.0035	16
GM					
-CH3	1	303.5	0	0.022	33.5
-CH2	4	269	0	0.02	16.1
-CH	20	176	0	0.013 (×2/3)	-1
-OH (prim.)	3	675	675	0.049	10
-OH (second.)	8	591	591	0.049	13
-O- (acetal)	4	236	102	0.018	3.8
-O- (acetal) in ring	4	236	102	0.018 (×2/3)	3.8
-COO-	1	640	528	0.05	18
6-membered ring	4	-48	61	-0.0035	16

Table S4. Printing parameters of representative polysaccharides.

Ink name	Concentration (wt%)	Nozzle Temp. (°C)	Pneumatic Pressure (kPa)	Printing Speed (mm s^{-1})
Agarose	8	80	300	10
Alginate	4	24	200	12
Chitosan	5	24	300	7
BSP	8	24	250	6-8
GM	4	24	350	10

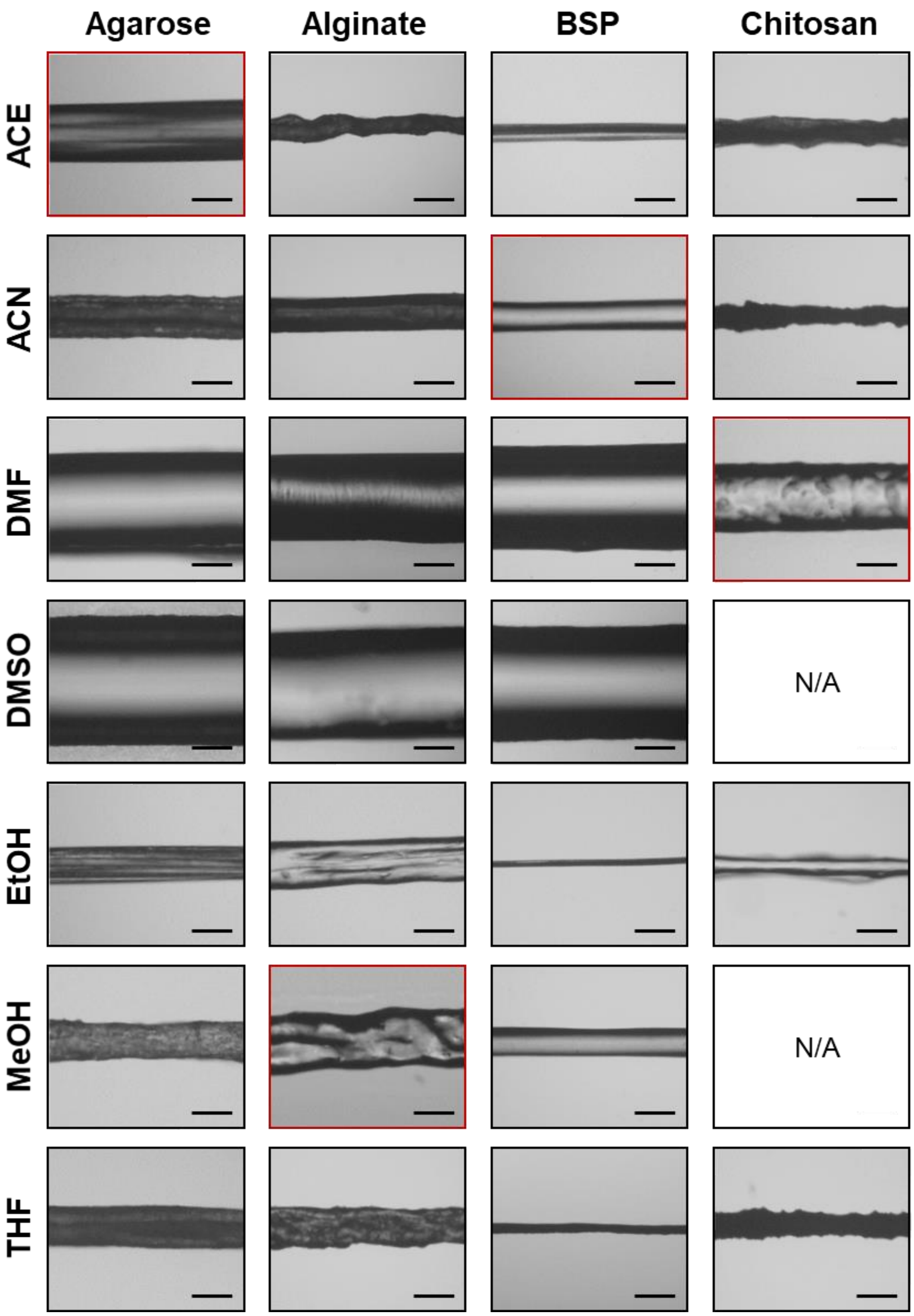

Figure S1. Filaments of natural polysaccharides formed in different non-solvents. Red frames: the optimal non-solvent for each polysaccharide. Scale bars: 250 μm.

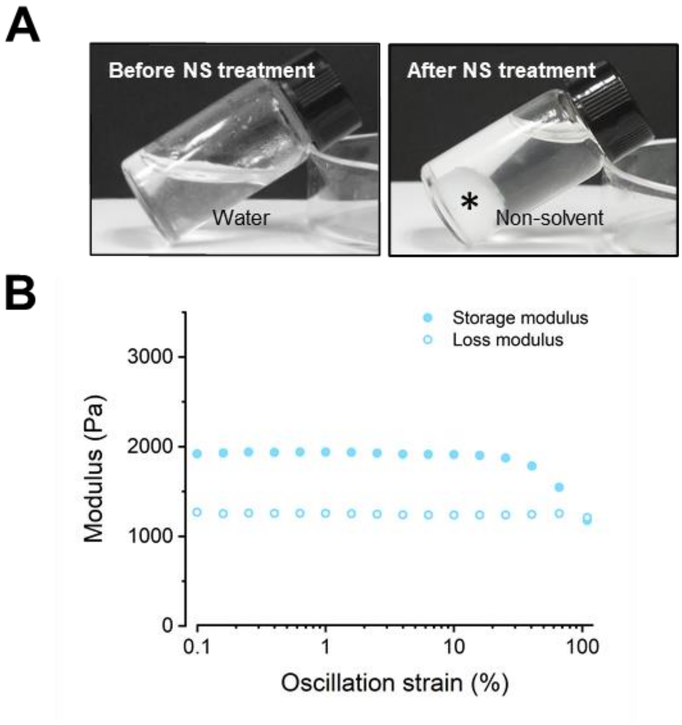

Figure S2. Mechanical enhancement of GM solution by non-solvent (NS) treatment. (A) Representative gross view of GM solution before and after NS treatment. NS: non-solvent. (B) Rheological characterization of GM solution after NS treatment, showing a remarkable increase from 187.7 Pa to 1921.17 Pa in the storage modulus. Asterisk, GM “organic gel”.

A

Elemental composition (%)			
Sample	N	C	H
CP-printed scaffold	0	1.12	8.043
NSQ-printed scaffold (before rinsing)	**8.48**	25.85	5.19
NSQ-printed scaffold (after rinsing)	0	0.82	7.685

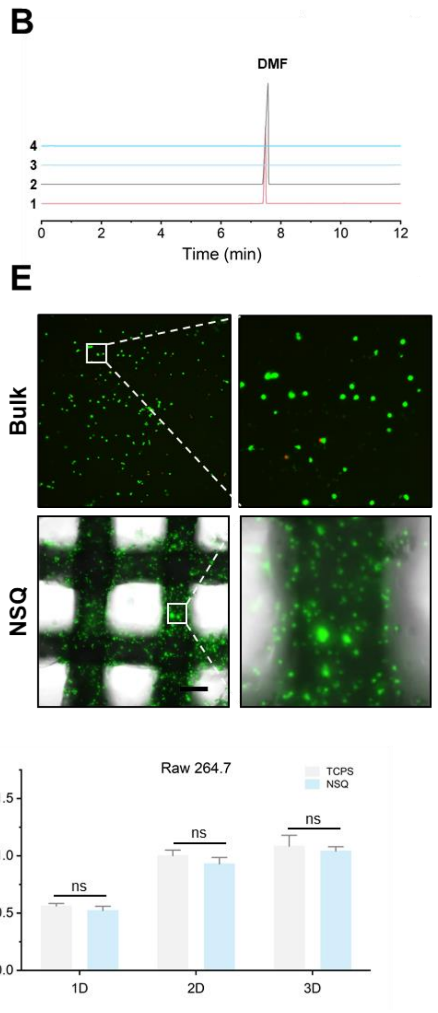

Figure S3. NSQ does not generate cytotoxicity. (A) Elemental composition and (B) GC chromatography of samples. 1: DMF; 2: CP-printed scaffold; 3: NSQ-printed scaffold (before rinsing); 4: NSQ-printed scaffold (after rinsing), showing effective removal of DMF from the scaffold after three rounds of thorough rinsing. (C) Live/dead images of HUVEC and Raw 264.7 cells cultured with medium extracting from GM scaffold at day 2 post seeding. (D) Cell viability for HUVEC and Raw 264.7 cells presented by the optical density values, as measured by CCK8 assay at days 1, 2 and 3 post seeding. ns, not significant (n = 6). (E) The adhesion of representative Raw 264.7 cells on Bulk hydrogel and NSQ scaffold. Scale bars: 200 μm in (C) and 100 μm in (E). Results are shown as mean ± SD. The differences between groups were analyzed using two-way repeated-measures ANOVA.

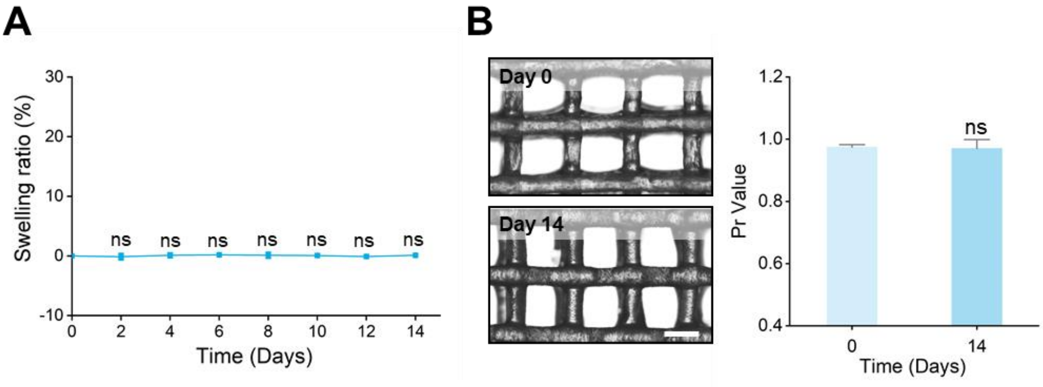

Figure S4. (A) The swelling ratio and (B) Pr values of GM scaffolds at day 0 and day 14. Inset images showed the scenarios of the grid constructs at two-time points. ns, not significant versus day 0 (n = 3). Scale bar: 500 µm. Results are shown as mean ± SD. The differences between groups were analyzed using a one-way repeated measures ANOVA.

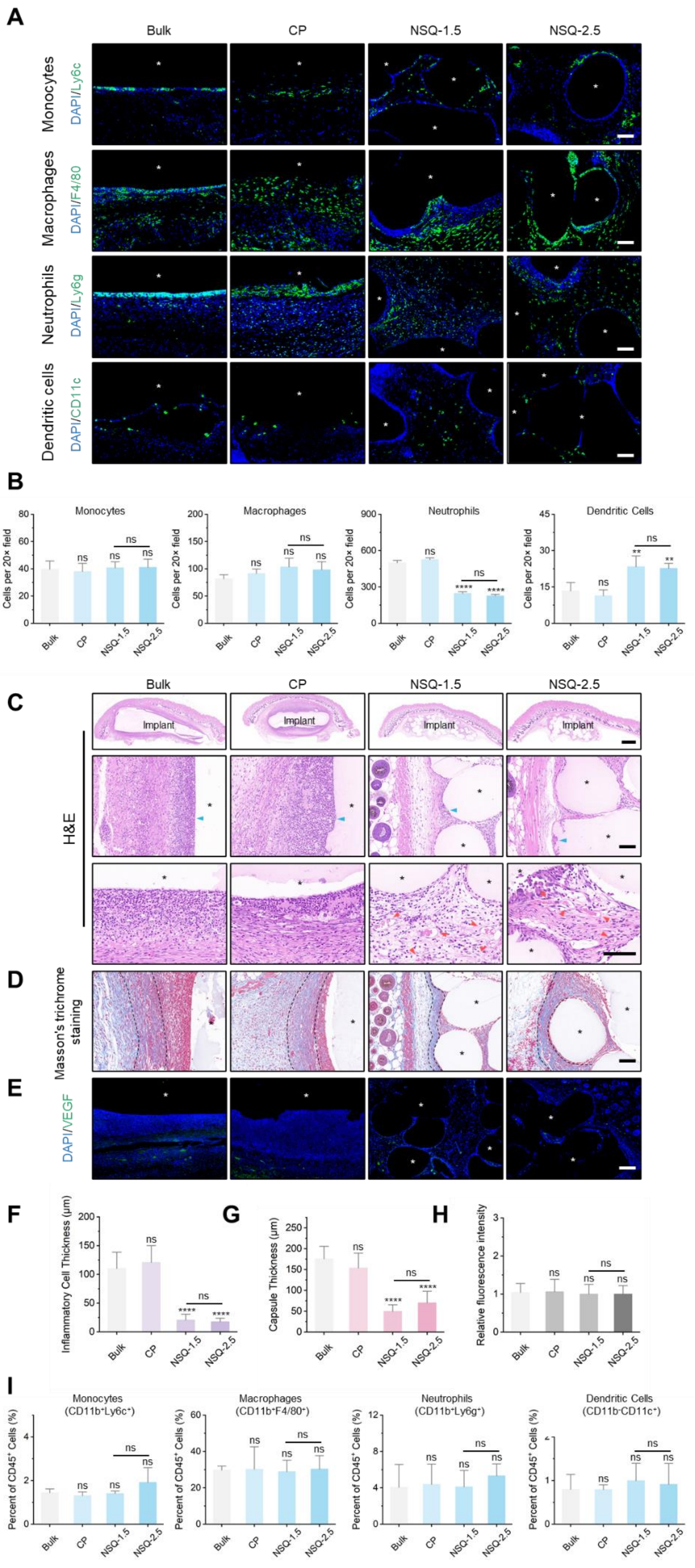
A
Bulk
CP
NSQ-1.5
NSQ-2.5
Monocytes
DAPI/Ly6c
Macrophages
DAPI/F4/80
Neutrophils
DAPI/Ly6g
Dendritic cells
DAPI/CD11c
B
Monocytes
Macrophages
Neutrophils
Dendritic Cells
Cells per 20× field
C
Implant
H&E
D
Masson's trichrome staining
E
DAPI/VEGF
F
Inflammatory Cell Thickness (μm)
G
Capsule Thickness (μm)
H
Relative fluorescence intensity
I
Monocytes (CD11b⁺Ly6c⁺)
Macrophages (CD11b⁺F4/80⁺)
Neutrophils (CD11b⁺Ly6g⁺)
Dendritic Cells (CD11b⁻CD11c⁺)
Percent of CD45⁺ Cells (%)

Figure S5. (A) Representative immunofluorescence staining for Ly6c (monocytes, green), F4/80 (macrophages, green), Ly6g (neutrophils, green), CD11c (Dendritic cells, green), and nuclei (4,6-diamidino-2-phenylindole [DAPI], blue). (B) Quantification of positive cell number per 20× field. $^{**}p < 0.01$; $^{****}p < 0.0001$; ns, not significant versus the bulk group (n = 3). (C) H&E staining. Blue arrowed: inflammatory cells; red arrowed: blood vessels. (D) Masson's trichrome staining. Dotted lines: thickness and location of fibrous capsule. (E) Immunofluorescence staining for VEGF (green) and nuclei (4,6-diamidino-2-phenylindole [DAPI], blue). (F) Quantification of the inflammatory cell thickness. $^{****}p < 0.0001$; ns, not significant versus the bulk group (n = 5). (G) Quantification of capsule thickness. $^{****}p < 0.0001$; ns, not significant versus the bulk group (n = 5). (H) Quantification of the relative fluorescence intensity. ns, not significant versus the bulk group (n = 3) (I) Flow cytometry of myeloid immune cell populations. ns, not significant versus the bulk group (n = 3). Scale bars: 100 μm in (A, D, and E) and 1 mm in (C) first row. Asterisk, implants. Results are shown as mean ± SD, with one-way ANOVA for statistical analysis.

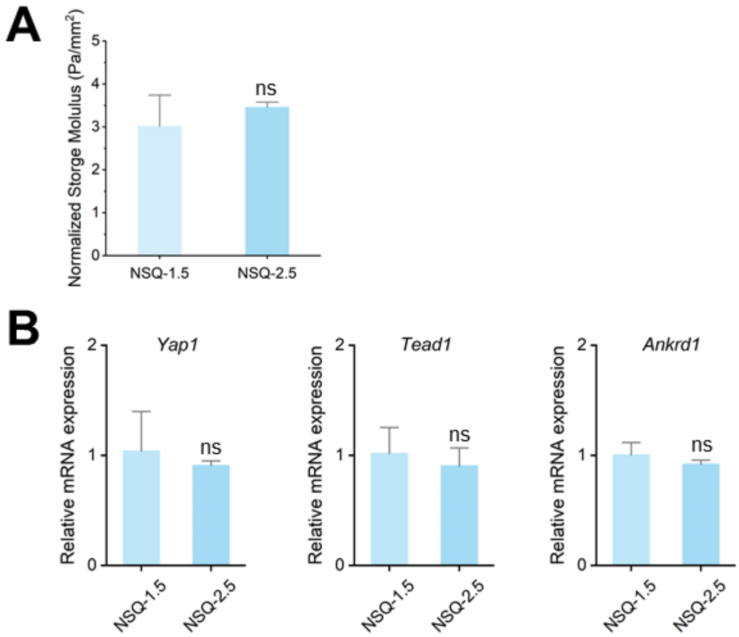

Figure S6. NSQ-1.5 and NSQ-2.5 have similar mechanical properties. (A) NSQ-1.5 and NSQ-2.5 showed no significant mechanical difference in microscopic level (normalized by surface area). (B) RT-qPCR analysis of the levels of representative YAP/TAZ target genes. ns, not significant versus the NSQ-1.5 group (n = 3). Results are shown as mean ± SD. The differences between groups were analyzed using an unpaired two-tailed *Student's t-test*.

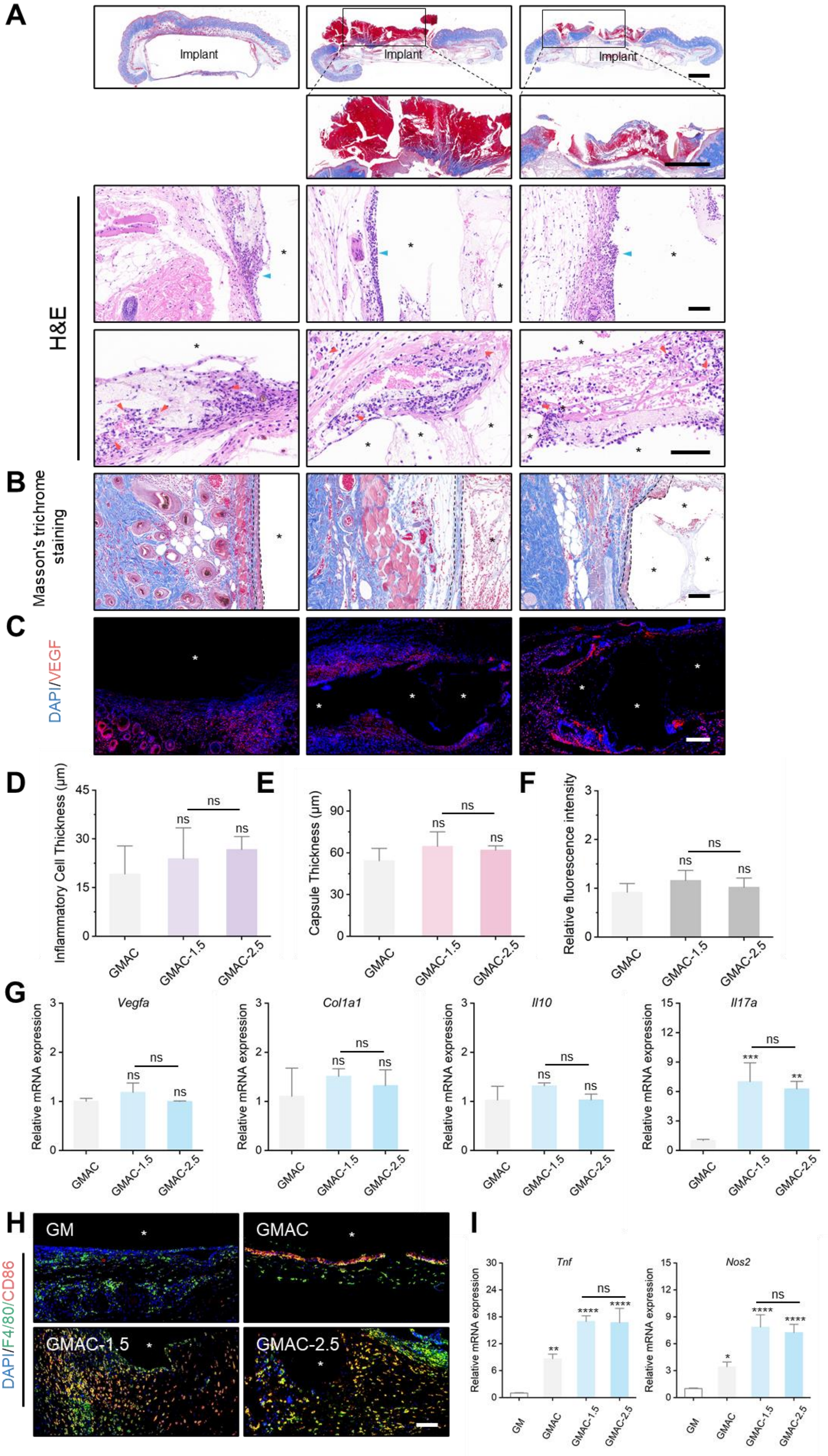
A
Implant
Implant
Implant
H&E
B
Masson's trichrome staining
C
DAPI/VEGF
D
Inflammatory Cell Thickness (μm)
GMAC
GMAC-1.5
GMAC-2.5
ns
E
Capsule Thickness (μm)
F
Relative fluorescence intensity
G
Relative mRNA expression
Vegfa
Col1a1
Il10
Il17a
***
**
H
DAPI/F4/80/CD86
GM
GMAC
GMAC-1.5
GMAC-2.5
I
Tnf
Nos2
****
*

Figure S7. Loss of subtle immunomodulation in 3D-printed acetyl GM scaffolds without using NSQ. (A) Representative gross view and H&E staining. Blue arrowed: inflammatory cells; red arrowed: blood vessels. (B) Masson's trichrome staining. Dotted lines: thickness and location of fibrous capsule. (C) Immunofluorescence staining for VEGF (red) and nuclei (4,6-diamidino-2-phenylindole [DAPI], blue). (D) Quantification of the inflammatory cell thickness. ns, not significant versus the GMAC group (n = 5). (E) Quantification of capsule thickness. ns, not significant versus the GMAC group (n = 5). (F) Quantification of the relative fluorescence intensity of VEGF. ns, not significant versus the GMAC group (n = 3). (G) RT-qPCR analysis of the levels of representative genes. $^{**}p < 0.01$; $^{***}p < 0.001$; ns: not significant versus the GMAC group (n=3). (H) Immunofluorescence staining for F4/80 (green), CD86 (red), and nuclei (4,6-diamidino-2-phenylindole [DAPI], blue). (I) RT-qPCR analysis of the levels of *Tnf* and *Nos2*. $^{*}p < 0.05$; $^{**}p < 0.01$; $^{****}p < 0.0001$ versus the GM group (n = 3). Scale bars: 1 mm in (A) first and second row, and 100 μm in (A, B, C, and H). Asterisk, implants. Results are shown as mean ± SD, with one-way ANOVA for statistical analysis.

Movie S1. Printing of GM via NSQ or CP, with NSP providing high fidelity and stability.

Please see the uploaded movie in the system.
